# Mitochondrial Dysfunction, Its Oxidative Stress-Induced Pathologies and Redox Bioregulation through Low-Dose Medical Ozone: A Systematic Review

**DOI:** 10.3390/molecules29122738

**Published:** 2024-06-08

**Authors:** Renate Viebahn-Haensler, Olga Sonia León Fernández

**Affiliations:** 1 Medical Society for the Use of Ozone in Prevention and Therapy, D-76473 Iffezheim, Germany; 2 Pharmacy and Food Institute, University of Havana, Calle 222 # 2317 e/23 y 31, Havana 10 400, Cuba

**Keywords:** redox bioregulation, mitochondriopathies, ozone therapy, oxidative stress, antioxidant capacity

## Abstract

Our hypothesis that controlled ozone applications interfere with the redox balance of a biological organism (first published in 1998 with a preclinical trial on protecting the liver from CCl_4_ intoxication) has been verified over the past two decades in reactive oxygen species (ROS)-induced mitochondrial pathologies, such as rheumatoid arthritis, osteoarthritis, aging processes and type 2 diabetes, and in the prevention of intoxications. Low-dose ozone acts as a redox bioregulator: the restoration of the disturbed redox balance is comprehensible in a number of preclinical and clinical studies by a remarkable increase in the antioxidant repair markers, here mainly shown as a glutathione increase and a reduction in oxidative stress markers, mainly malondialdehyde. The mechanism of action is shown, and relevant data are displayed, evaluated and comprehensively discussed: the repair side of the equilibrium increases by 21% up to 140% compared to the non-ozone-treated groups and depending on the indication, the stress markers are simultaneously reduced, and the redox system regains its balance.

## 1. Introduction

As the powerhouse of cells, mitochondria have the essential function and task of maintaining cellular metabolic balance: the provision of energy in the form of ATP (adenosine triphosphate) plays a central role as well as the production of reactive oxygen species (ROS) and their protective antioxidants in order to maintain the redox balance.

A small part of energy is provided by the citrate cycle, in which “all” substances ingested with food are catabolized: fats, carbohydrates and proteins. In the matrix space of the mitochondrion, the citrate cycle is in close proximity to the enzyme complexes of oxidative phosphorylation, to which the reduction equivalents formed here are made available for the exergonic redox reactions. The lion’s share of ATP comes from the respiratory chain and oxidative phosphorylation (36 mol ATP/mol glucose), which is extremely dependent on an optimal oxygen supply due to the high oxygen requirement.

Defects in mitochondrial energy metabolism can lead to a variety of diseases, be they of a genetic origin, defects in mitochondrial DNA (mtDNA) or due to the normal aging process, or an accumulation of mitochondrial defects that result in increasing ATP deficiency and an increased production of reactive oxygen species (ROS) [1,2,3].

We here focus on diseases that are causally linked to high oxidative stress, the overproduction of superoxide radicals and corresponding secondary products and the ROS, and lead to a disturbance of the mitochondrial and cellular redox balance.

### 1.1. Mitochondrial ROS Production

Among the enzyme complexes, located in the inner mitochondrial membrane, complex IV is mainly responsible for the formation of superoxide radicals, O_2_^−^.

In a healthy, energetically intact system, the heme centers of the cytochrome complexes (Fe^2+^→Fe^3+^ + e^−^) transfer two electrons per mole of oxygen, so that O_2_ is formally reduced to O^2−^ or H_2_O. In the case of mitochondrial insufficiency, the electron transfer is also deficient, and oxygen reduction stops with a one-electron transfer, forming O_2_^−^ [1]. Among the ROS subsequently formed, H_2_O_2_ and the ⋅OH radicals have a special function: essential for the defense against infections, H_2_O_2_ also acts as an important signal and regulatory molecule [2,3].

The mitochondria’s own antioxidant system—superoxide dismutase-2 (SOD2), catalase (CAT), glutathione peroxidase (GSHox), glutathione reductase (GSred) and others—responsible for maintaining the mitochondrial redox balance, also deals with external oxidative stress, thus forming a cellular ROS sink to a certain extent [4]. If the antioxidant system is overwhelmed, the redox balance shifts in favor of an overproduction of reactive oxygen species, with ·OH radicals ultimately being responsible for the degenerative processes due to their non-specific and high reactivity. Enzymes are blocked, and metabolic pathways are slowed down, e.g., the citrate cycle. ATP production decreases and the cell switches its survival strategy to other metabolic pathways, such as anaerobic glycolysis in the cytoplasm (Warburg effect), which leads to neoplastic cells [5,6]. Mitochondrial dysfunction can therefore induce a variety of diseases, cancer, vascular inflammatory processes and associated diseases, age-related diseases or neurodegenerative processes, as summarized in Table 1.

### 1.2. Mitochondriopathies, Preferred Indications for Redox Regulation through Ozone

The low-dose ozone concept with its redox regulatory effect has emerged as a successful complementary treatment strategy in mitochondriopathies linked to high oxidative stress as listed in Table 2 (without any claim to completeness). We here only refer to diseases based on clinical studies and animal trials as published in peer-reviewed journals. Our focus is on a disturbed and restored redox balance, illustrated by very few typical parameters to demonstrate the common basis: oxidative stress versus the repair effect obtained by redox regulation through ozone.

## 2. Evaluation and Discussion of the Relevant Data in Redox Regulation for Selected Mitochondriopathies

**Figure 1:**
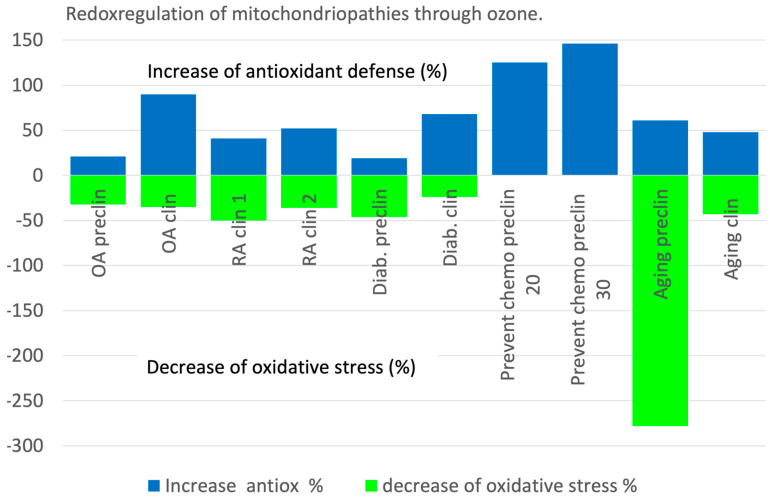
Redox bioregulation in ROS-induced mitochondriopathies through ozone: the restoration of the redox balance is verifiable in all preclinical and clinical studies; only those in which no redox parameters were available could not be included. Increases in antioxidant defense measured as GSH in % compared to the non-ozone-treated controls and decrease in oxidative stress compared to the non-ozone-treated controls, expressed as MDA (TH only in diabetes clin.) in %. For details, see text. OA preclin: Osteoarthritis in an animal study; OA clin: Osteoarthritis in a clinical study; RA clin: Rheumatoid arthritis, clinical study; Diab preclin: Animal study with STZ-induced diabetes; Diab clin: Clinical trial in type 2 diabetes; Prevent chemo preclin: Prevention of kidney damage due to cis platinum intoxication by rectal ozone application with an ozone concentration of 20 µg/mL and 30 µg/mL; Aging preclin: Animal trial in aging by lifelong regular rectal ozone application; Aging clin: Clinical study in attenuating the aging process.

Our hypothesis that controlled ozone applications interfere with the redox balance of a biological organism, first published in 1998 [26] with a preclinical trial in protecting the liver from CCl_4_ intoxication, has been verified over the past two decades. In ROS-induced mitochondriopathies, low-dose ozone acts as a redox bioregulator: a restoration of the redox balance is verifiable in all preclinical and clinical studies—those in which no redox parameters were available have not been included in this paper. Here, we find the basic mechanism of action of systemic ozone applications, which, in addition to the known mechanisms of disinfection in topical ozone application (wound cleansing and wound disinfection), covers all ozone indications: chronic inflammatory diseases, silent inflammation and diseases associated with high oxidative stress—antioxidants as repair and protecting markers increase and stress markers decrease as discussed and summarized in Figure 1.

The repair side of the equilibrium increases by 21 up to 140% compared with the non-ozone-treated groups, and the stress markers are simultaneously reduced by at least 24% to approx. 50%, in the aging process using an animal model, even to the extent of 278%.

### 2.1. Reference Substances

To verify the regulatory effect of medical ozone in mitochondrial pathologies, significant, reliable and reproducible oxidative stress parameters were measured, MDA, TH and corresponding parameters from the group of antioxidants SOD, CAT, GSHox, GSred and others in order to recognize the restoration of the redox balance. As all of these diseases are accompanied by a GSH deficiency (reduced glutathione), it makes particular sense to measure GSH as a follow-up parameter. For each study, we here discuss the corresponding reference substances; see also Table 3.

### 2.2. Toxicity of Ozone versus Therapeutical Efficacy

Over the past two decades, a number of preclinical trials, confirmed in clinical studies, have revealed the effective, non-toxic concentrations and doses of medical ozone and have allowed us to set up the guidelines for the use of ozone in prevention and therapy [27].

Ozone concentrations in the range of 10 to 30 µg/mL (max. 40 µg/mL) are those preferred for systemic application and the redox bioregulatory effect, taking a volume of 50–100 mL in the case of extracorporeal blood treatment (major autohemotherapy, MAH) or 200–300 mL in the case of rectal insufflation (RI). High concentrations contribute to oxidative stress blocking the regulation. They are administered locally to make use of the germicidal effect of ozone in highly infected wounds. For wound healing, the concentrations have to be reduced drastically to the systemic ones. High concentrations would destroy the freshly formed epithelium; the wounds would enlarge and would never heal [28].

### 2.3. Evaluation of Mitochondrial Pathologies and the Regulatory Effect of Medical Ozone: Restoration of the Redox Balance

Frequently and successfully applied in private clinics to avoid surgical interventions, preclinical and clinical studies in knee osteoarthritis were not published before 2010. Since then, however, a large number of clinical studies have been carried out. Our aim here is to demonstrate the rationale, namely to regulate the redox balance that has grown out of control, by administering ozone systemically or locally. For this purpose, a few exemplary redox parameters are analyzed in preclinical and clinical studies; see Table 2.

#### 2.3.1. Osteoarthritis

**Figure 2:**
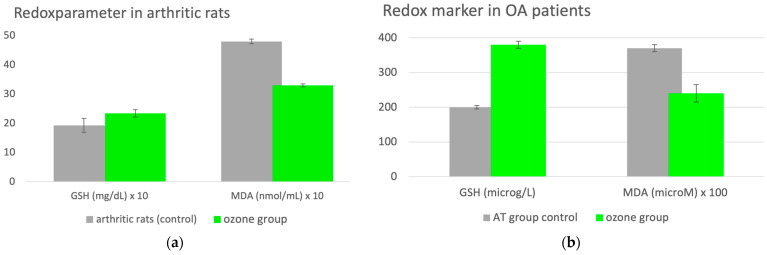
(**a**). Redox regulation with ozone in a knee arthritis model in rats (preventive arm of the study). GSH as the most important non-enzymatic antioxidant in the cells is a sensitive parameter to be followed during the treatment of inflammation, whereby it is reduced in inflammatory processes (81% of the healthy controls; please see original publication [12]). Here, GSH of arthritic rats without ozone is taken as a control. Ozone was administered as follows: 15 rectal insufflations (5 per week) at a dose of 0.5–0.7–1.0 mg ozone per kg body weight, increasing from week 1 to 3, and a corresponding volume from 5–6 mL; GSH increases to healthy values. As a consequence, the oxidative stress, here as MDA, decreases by 32% to normal values (*p* < 0.05) and the redox balance is restored [12]. (**b**). Redox regulation through systemic, preventive ozone treatment in 40 patients with knee osteoarthritis undergoing a surgical procedure by arthroscopy. The AT group (n = 20) without ozone and ozone group (ozone + AT), n = 20. Ozone application: 20 rectal insufflations; concentrations, dose and treatment frequency: 25–35 increasing from week 1 to week 4, volume at 150–200 mL, 5×/week. Measurement (plasma) of antioxidant status as GSH and oxidative stress as MDA 30 days after surgical intervention. Ozone preconditioning was able to restore the redox balance, antioxidants were upregulated, GSH increased by 89% and oxidative stress, here as MDA, decreased by 35% (*p* < 0.05): intra-articular ozone modulates inflammation [13].

A preclinical trial in an animal model with rats [12] using only one parameter on the oxidative stress side of the balance, one of the main causes of the disease, and one of the antioxidants on the other side: MDA and GSH, very reliable and reproducible, were used as reference substances representing the effect of ozone. The study was designed for two study arms, firstly a preventive arm in which ozone was administered prior to inducing arthritis in the animals and secondly the therapeutic arm ozone was administered after the arthritis had been manifesting for 24 days. The route of ozone administration was exactly the same and—interestingly enough—the results were the same: an increase in antioxidative capacity, here measured as GSH, returned to normal values as well as oxidative stress, here, MDA. This means the following: systemically administered ozone as a preventive (protective) or therapeutic measure is able to restore redox balance in inflammatory diseases, in this case, knee arthritis (Figure 2a).

Clinical trial—systemic ozone regulates the redox balance in patients with osteoarthritis: A beneficial, preconditioning effect of ozone in patients with osteoarthritis was revealed in a clinical study involving 40 patients undergoing a surgical procedure via arthroscopy (AT). A total of 20 patients received ozone in the form of rectal insufflation before AT (ozone group) and 20 underwent arthroscopy without the preventive ozone measure (AT group). Clinical and biochemical parameters were determined 30 days later (for the whole spectrum, material and methods, please see original publication). Here, again, we focus on the redox balance and evaluate GSH as an antioxidant and MDA as an oxidative stress parameter, shown in Figure 2b. GSH as a protective redox biomarker is upregulated by 85% through ozone application prior to arthroscopy. MDA as an injury redox biomarker decreases accordingly by 35%, corresponding to a restoration of the redox balance [13].

**Figure 3:**
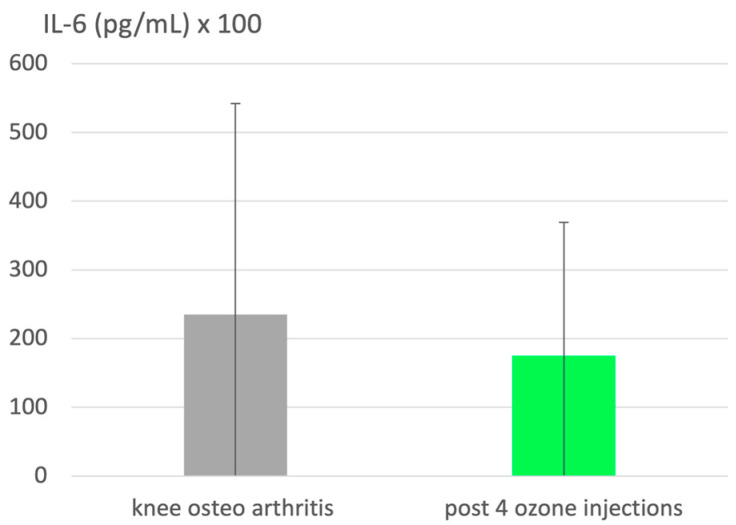
A clinical trial (n = 51) in patients with knee osteoarthritis demonstrates the anti-inflammatory effect of ozone on IL-6, a pro-inflammatory cytokine, typically increased in inflammatory processes. IL-6 is downregulated by 26% following 4 intra-articular ozone infiltrations (*p* = 0.0697). Procedure: 20 mL ozone at a concentration of 20 µg/mL. Clinical markers and other biochemical parameters correspond to the improvement in inflammatory conditions of the disease: please consult the original publication [14].

The upregulation of antioxidants and downregulation of pro-inflammatory cytokines, such as IL-1, IL-6 and TNF-α, in inflammatory diseases is well known. In this context, a clinical study on knee osteoarthritis [14] comprising 51 patients (non-diabetic, non-obesity) is taken to demonstrate the anti-inflammatory effect of ozone in knee osteoarthritis as an example of high oxidative stress in mitochondrial deficiencies. Elevated IL-6 serves as a typical parameter. It is downregulated by 26% (Figure 3), simultaneously improving the clinical parameters. The ozone mechanism of action involving the nuclear factor NFkB is discussed in Section 4 of this article.

#### 2.3.2. Rheumatoid Arthritis (RA)

Torequl Islam et al. (2023) [29] published a comprehensive overview on the pathogenesis of rheumatoid diseases, the dysregulated redox system and therapeutic strategies including complementary therapies.

Ozone improves cellular redox balance in patients with rheumatoid arthritis.

Here, we focus again on the critical parameters GSH and MDA and the restoration of the redox balance to show and underline the basic mechanism of ozone in mitochondriopathies.

**Figure 4:**
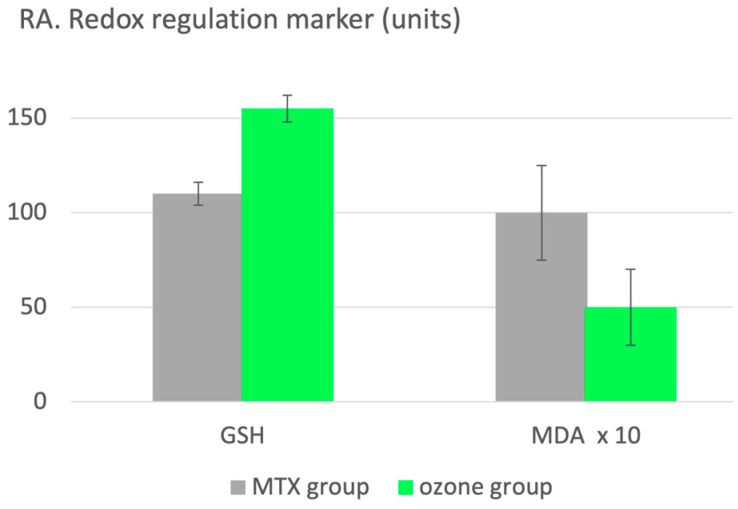
Redox regulation in patients with rheumatic arthritis using the low-dose ozone concept. Two groups: the MTX group (n = 30) received the basic therapy only, methotrexate, ibuprofen and folic acid, and here serves as the 100% control. The ozone group (n = 30), applying the same basic therapy, received ozone as a complement. GSH increases by 41% and the oxidative stress parameter decreases by 50%; *p* < 0.05 [15].

A total of 60 patients with RA received a basic therapy: methotrexate (MTX) + ibuprofen + folic acid. The MTX group (n = 30) received basic therapy only and the ozone group (n = 30) received the same treatment as the MTX group plus additional ozone. Ozone treatment: 20 rectal insufflations. The concentration was 25–40 µg/mL, increasing by 5 µg/mL per week with a volume of 150–200 mL during 4 weeks. As per usual in inflammatory processes, the redox balance is regulated through systemically administered ozone at low concentrations and low doses, demonstrated by the characteristic increase in the antioxidant defense, shown in Figure 4 as an increase in GSH by 41% and a decrease in oxidative stress by 50% as a consequence, here, MDA [15].

##### A Redox Regulator with Selectivity for Patients with Rheumatoid Arthritis

Although RA and osteoarthritis are of a different origin, both are compared concerning the impact of redox regulation through ozone. RA as an autoimmune disease is usually treated using antirheumatic drugs, such as MTX, which involves a risk of liver toxicity. In patients with osteoarthritis, the therapy is mostly based on nonsteroidal anti-inflammatory drugs (NSAIDs). Combined therapy with ozone at low concentrations has proven its worth in RA as well as in OA [17].

Rheumatoid arthritis: A controlled clinical study, n = 40, involved basic therapy—MTX, ibuprofen and folic acid. The MTX group as a control, n = 20, received basic therapy only. The ozone group, n = 20, received the same basic therapy and additional ozone application.

Osteoarthritis: A controlled clinical study, n = 40, involved basic therapy—NSAIDs. The control, n = 20, received basic therapy only. The ozone group received basic therapy plus ozone application as described above.

Apart from the clinical parameters, such as pain and disability, the redox balance was also assessed using MDA, TH and GGT as injury markers and GSH, SOD and CAT as antioxidant defense markers. Here, we once more discuss GSH and MDA: in the patients with RA, the antioxidant capacity, measured as GSH, increases by 25% and the oxidative stress—here as MDA—decreases by 31%. Thus, the redox balance is largely restored in rheumatoid arthritis.

**Figure 5:**
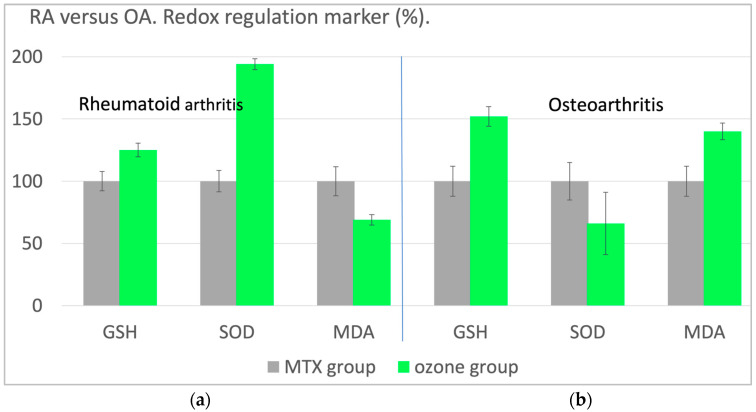
Redox regulation through systemically administered ozone. (**a**). Rheumatoid arthritis: Controlled clinical study—n = 40: MTX group (MTX, ibuprofen, folic acid) serves as a control; n = 20 with basic therapy only. The ozone group, n = 20, received the same basic therapy and additional ozone application. (**b**). Osteoarthritis: Controlled clinical study—n = 40: NSAIDs as basic therapy (control) and n = 20 with basic therapy only. The ozone group received basic therapy plus rectal ozone application as described above. Antioxidants, GSH and SOD here, increased in RA by 25% or 94%, respectively. In OA, there was an increase in GSH by 52% although a drastic decrease in SOD was measured, and oxidative stress—MDA here—showed a remarkable rise. Result: RA responds to the systemic ozone application to regulate and to restore the redox balance, which is not so in the case of OA; oxidative stress even increases by 40% [17].

In comparison, ozone application is less successful in the osteoarthritis trial. Although GSH increases by 52% (whereby the absolute values are considerably lower compared to RA), oxidative stress also increases. With SOD as a typical antioxidant, the effect is much more pronounced. SOD even decreases by approx. 34% in OA. Superoxide radicals cannot be reduced, which explains the increase in the oxidative stress parameter MDA. Obviously, a restoration of the disturbed redox balance cannot be achieved in osteoarthritis (Figure 5).

##### Medical Ozone Effects and Innate Immune Memory

This study in patients with RA (n = 20) shows the same results as those revealed by León et al. (2016) [15], and even better results after a second cycle of ozone treatment due to the formation of memory cells during or after the first treatment cycle [16].

#### 2.3.3. Diabetes

Preclinical study: Ozone treatment regulates oxidative stress in an experimental diabetes model in rats, shown in STZ-induced diabetes.

Since diabetes is closely associated with oxidative stress, this first animal model in diabetes with ozone allows us to confirm the hypothesis that medical ozone is able to regulate and/or restore the redox balance. The procedure was rectal insufflation with an ozone amount of 1.1 mg/kg rat, and 10 treatments in 2 weeks.

**Figure 6:**
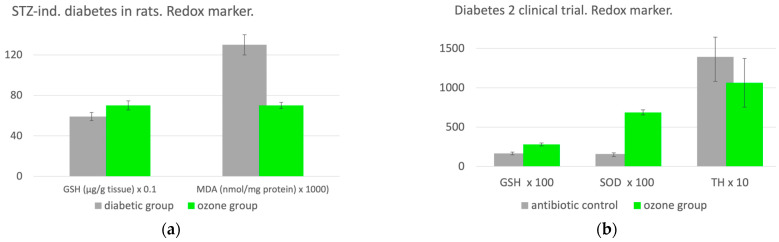
(**a**). Redox regulation by ozone in a diabetes animal model. The preclinical trial with STZ-induced diabetes in rats, n = 50. As diabetes is closely associated with oxidative stress, this animal model allows us to confirm the hypothesis that medical ozone is able to regulate and/or restore redox balance. Procedure: Rectal insufflation with ozone at 1.1 mg/kg rat, and 10 treatments over 2 weeks. GSH as a repair parameter increases by 19% after ozone treatment and the oxidative stress parameter drops by 46%, thus demonstrating a pronounced regulatory process [18]. (**b**). The clinical study in patients with diabetes and diabetic gangrene. The antibiotic group, n = 49, received antibiotic infusions according to the specific germs. The patients in the ozone group, n = 51, were treated with 20 rectal ozone insufflations, 5 per week, applying a volume of 200 mL and an ozone concentration of 50 µg/mL. The healing process improved, and plasma glucose decreased. Total hydroperoxides as an oxidative stress parameter decreased, corresponding to an increase in SOD, which could not be seen in MDA. GSH and TH were measured in µMol, and SOD in U/mL/min; *p* < 0.05 [19].

Out of five animal groups (10 in each group), the diabetic animal group serves as a control. Redox regulation is clearly pronounced: GSH increases by 19% after ozone application, and oxidative stress drops by 46% as presented in Figure 6a [18].

Diabetes, clinical study: Therapeutic efficacy of ozone in patients with a diabetic foot—in type 2 diabetes, oxidative stress not only plays a key role in the development of the disease, but also to a particular extent in the progression of metabolic disorders, angiopathies and typical concomitant diseases. In this clinical study, 101 patients with a diabetic foot were examined and the clinical findings as well as the metabolic results under systemic ozone administration were analyzed. An antibiotic group with systemic antibiotics and conventional wound care served as a control, n = 49. A total of 51 patients in the ozone group received five ozone treatments per week within 4 weeks in the form of rectal insufflation, taking a volume of 200 mL and an ozone concentration of 50 µg/mL. The healing process improved, and plasma glucose decreased. Total hydroperoxides as an oxidative stress parameter decreased, corresponding to an increase in SOD, which could not be seen in MDA. The results are displayed in Figure 6b [19].

#### 2.3.4. Oncology: Prevention of Side Effects of Chemotherapy

**Figure 7:**
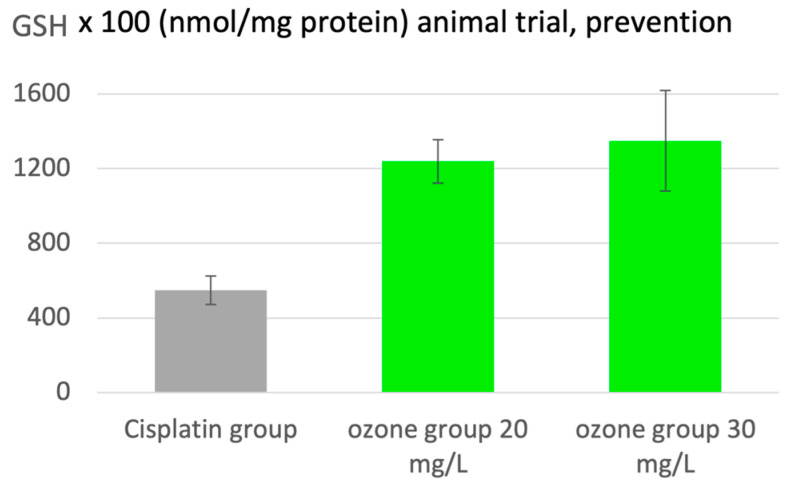
Protection by ozone preconditioning in cisplatin-induced nephrotoxicity in rats. A total of 15 rectal insufflations in 3 weeks prior to cisplatin. A total of 10 groups with 7 animals each; here, the cisplatin group is a control and there are 2 ozone groups, ozone conc. 20 and 30 µg/mL. Ozone pretreatment protects from nephrotoxicity and keeps GSH within the healthy range (non-treated controls), but only with ozone concentrations below 50 µg/mL; *p* < 0.05 [23].

In oncology, ozone can be used as a part of a complementary therapeutic system, and its major effect is a protective one, the prevention of liver and kidney intoxication from chemotherapeutics, here shown in an animal model with kidney intoxication through cisplatin. Ozone interferes by the upregulation of the antioxidants and downregulation of oxidative stress, as shown in Figure 7 with GSH as a sensitive parameter in redox regulation. At low ozone concentrations (20 to 30 µg/mL), kidney GSH remains within the healthy range after cisplatin administration, but not at concentrations of 50 µg/mL or 70 µg/mL, where ozone itself contributes to toxicity. A preclinical trial with 70 rats in 10 groups demonstrates the protective effect; ozone was administered via rectal insufflation, with approximately 7 mL and ozone at 0.7 to 1.0 mg/kg rat corresponding to an ozone concentration of 20 to 30 µg/mL: the redox balance remains completely intact [23]. These low ozone concentrations do not induce cancer cell proliferation [30], but the concentration-dependent influence on Nrf2 is an intensive topic in other working groups, and requires further investigation and is reserved for later publications [31].

#### 2.3.5. Aging

We cannot stop the aging process, but we can influence high oxidative stress as one of its main causes due to mitochondrial aging and dysfunction. Low-dose ozone applications are able to intervene here as a redox regulator and offer at least one preventive measure against age-related diseases.

Preclinical trial: Shehata et al. [32] and Safwat et al. [24] set up preclinical studies in rats demonstrating a significant regulation of the disturbed redox balance by systemic ozone administration in the form of rectal insufflation. The critical redox parameters GSH and MDA (measured in the liver here) and others underline the restoration of the balance by life-long preventive as well as by “therapeutic” (not shown here) treatments in older animals. GSH and MDA are expressed in % of healthy adult animals aged 4 months. GSH antioxidant capacity decreases in the aging process by 25% (*p* < 0.001), and oxidative stress increases by 280%. The redox balance is manifestly disturbed.

**Figure 8:**
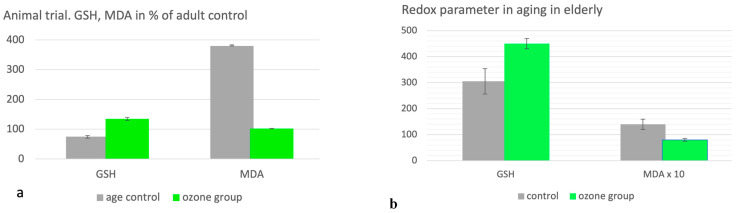
(**a**). Ozone in aging. Animal study on prevention. Age control: Healthy, normal-aging animals at 14 months of age. Ozone group: Rectal ozone application regularly from month 4 to month 14 (3 × per week; later, 2 × per week; ozone concentration: 20 mg/L; and volume of ca. 5 mL, corresponding to 0.6 mg/kg body weight). Redox parameter: GSH as antioxidant capacity and MDA as oxidative stress marker displayed as percentage from healthy adults, 4-month-old animals and adult control [24,32]. (**b**). Development of redox parameters GSH and MDA (in µM) in elderly patients with comorbidities rheumatoid arthritis and diabetes (n = 30). Control (n = 15): After 4 weeks of basic treatment only. Ozone group: After 4 weeks of basic treatments plus ozone. Ozone administration: 20 rectal insufflations, volume: 200 mL and ozone concentrations: 20 to 30 µg/mL, increasing from week 1 to 4. GSH shows level 48% higher after 4 weeks compared with control group, *p* < 0.05. MDA is 43% lower than in control group without ozone treatment, *p* < 0.05 [25].

Preventive ozone administration was able to increase GSH by 25% compared with the healthy control and 61% to untreated, normally aging animals (age control). Oxidative stress in the form of MDA increased accordingly: age control, untreated animals to 380%, and ozone group to 102%, which is 278% less than the normally aging animals (Figure 8a), *p* < 0.001.

##### Clinical Study in the Elderly

Elderly patients at the age of 60 to 75 years with diabetes and rheumatoid arthritis as comorbidities were examined in a controlled, randomized clinical study. All of them received a basic therapy. The control (n = 15) received the basic medication only, and the ozone group (n = 15) received basic medication plus ozone treatment. The ozone concept was 20 rectal insufflations over 4 weeks and 5 per week. Concentrations were 20 up to 30 µg/mL, slowly increasing week by week. A third group with asthma (n = 30) is not discussed here.

As a redox parameter, we once more use GSH as a defense marker and MDA as an oxidative stress parameter after 4 weeks of treatment without and with ozone.

Compared within the baseline (start at 450 µM, GSH dropped by 67% in the control group after 4 weeks with basic treatment only, whereas GSH showed no change in the ozone group (*p* < 0.05).

Ozone treatment attenuates the aging process, thus preventing from age-related diseases: GSH shows a 48% higher level after 4 weeks compared with the control group. As a consequence of the upregulation of antioxidants through ozone, the oxidative stress, measured as MDA, is 43% lower than in the control group without ozone treatment, *p* < 0.05; see Figure 8b [25].

#### 2.3.6. Prevention

**Figure 9:**
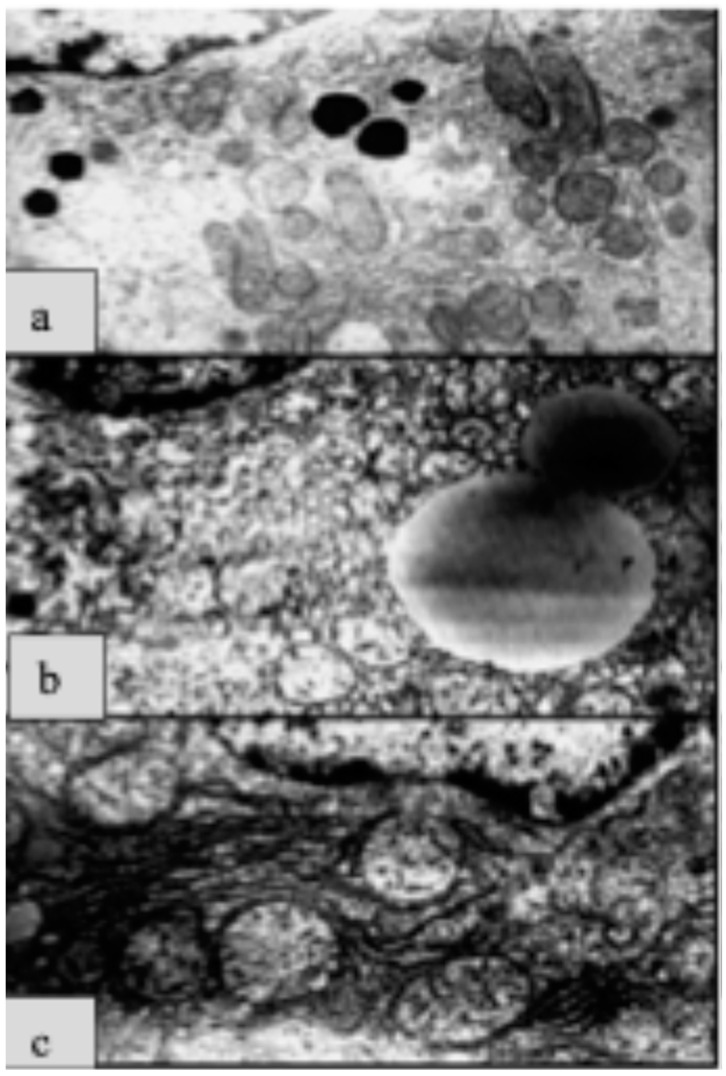
Histological lesions in liver tissue after reperfusion damage. (**a**) Control: Regular structure of the mitochondria and other organelles. (**b**) Large lipid droplets and moderate reperfusion damage are visible after I/R (90 min ischemia and 90 min reperfusion), while ozone preconditioning (**c**) causes extensive integrity of the mitochondria [34].

Preventive, therapeutic measures are always difficult to communicate to patients, but in the wake of pandemics, people tend to be interested in and open to prevention, especially in the case of impending viral infections. This is where the use of medical ozone has proven to be useful [33], supported by a number of animal models, but also clinical studies, as discussed above. It has been repeatedly shown that the liver as a detoxification organ responds particularly well to ozone, so that intoxications caused by drugs such as MTX [15], chemotherapeutics [23] and others can be largely avoided. Preventive administration prior to surgical interventions, for example, to avoid reperfusion damage, which is the rule in transplantations, appears to be particularly reasonable [34]. In addition to the restoration of the redox balance (GSH increase and MDA decrease—among other characteristic parameters), electron microscopic examinations show in animal experiments that the integrity of the mitochondria is largely preserved by ozone preconditioning (OP); see Figure 9: in Figure 9a, the regular structure of the mitochondria and other organelles in liver tissue can be seen. In Figure 9b, large lipid droplets and moderate reperfusion damage are visible after I/R (90 min ischemia and 90 min reperfusion), while OP causes extensive integrity of the mitochondria (Figure 9c).

## 3. Mechanism of Action

**Figure 10:**
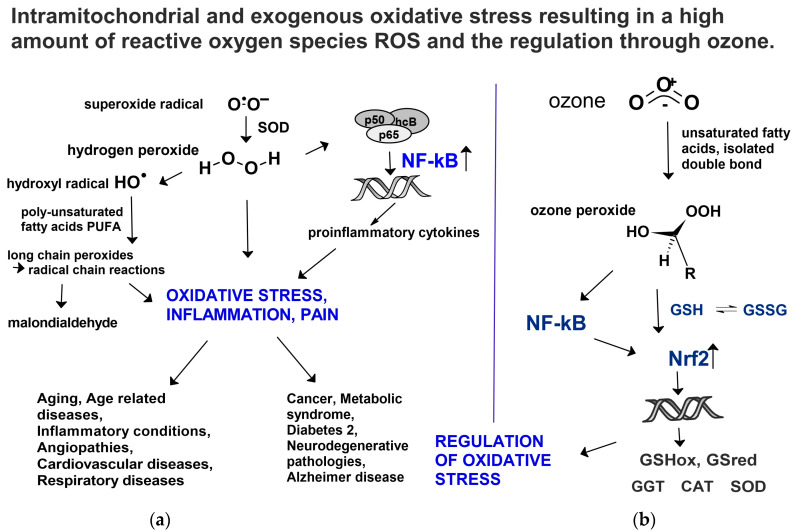
Mitochondrial and exogenous oxidative stress and its regulation via ozone. (**a**) Mitochondrial deficiency with formation of superoxide radicals: one electron transfer to oxygen forming in ⋅O-O^−^, superoxide radical as starting molecule for ROS production, such as H_2_O_2_, ⋅OH radicals, long-chain peroxides and others, inducing mitochondrial pathologies. (**b**) “Ozone peroxide” reduced by GSH, informing DNA via NFkB and Nrf2 upregulating antioxidants in order to restore unbalanced ROS/antioxidant equilibrium. GSHox: Glutathione peroxidase, GSred: Glutathione reductase, GGT: γ-Glutamyl transferase, CAT: Catalase, SOD: Superoxide dismutase, NFkB and Nrf2: Nuclear factors.

The anti-inflammatory and regulatory mechanism of ozone was described in detail in an earlier paper [33] and is summarized here in Figure 10: an excessive production of ROS, mostly as a result of mitochondria deficiency, leads to high oxidative stress, inflammation and a number of ROS-based diseases, such as chronic inflammation, rheumatoid arthritis, cerebral and peripheral angiopathies, metabolic syndrome and type 2 diabetes, cardiovascular diseases, cancer, neurodegenerative pathologies and others.

In general, ozone or rather second messenger “ozone peroxide” as a reaction product interferes via the glutathione balance GSH/GSSG by signal transduction, Nrf2 activation and translocation to the nucleus.

**Figure 11:**
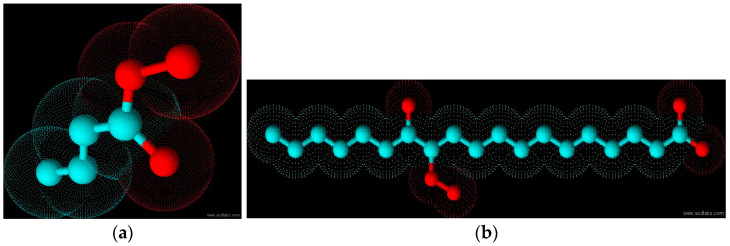
(**a**). Ozone peroxide, molecular model. High reactivity with terminal peroxide group and OH group (red) at same terminal C atom. (**b**). Molecular model: Long-living peroxide. Contribution to high oxidative stress with central peroxide group and OH (red) at different C atoms (green), inducing radical chain reactions.

Ozone peroxides at low concentrations and low doses do not contribute to oxidative stress in the entire system as we are dealing with a small and reactive molecule having a short half-life, completely different from the long-chain peroxides (Figure 11a,b), which are stable and have a fairly long half-life, inducing radical chain reactions and contributing to chronic oxidative stress.

Following an activation of NFkB and a cross-talk to Nrf2 [35,36], antioxidants are upregulated and available to reduce the ROS in order to at least partially restore the unbalanced ROS/antioxidant equilibrium.

## 4. Conclusions and Future Perspectives

Since medical ozone as a redox bioregulator is well established in the international literature, we understand the typical ozone indications much better as well as the integration into a complementary system, either in prevention or therapy. ROS-induced mitochondriopathies are the major part of these indications: the disturbed redox balance is regulated by the low-concentration and low-dose ozone concept. It is time to and urgently needed to enable larger clinical trials, as they already exist in pain therapy with several thousand patients (among others, [37,38]).

## Figures and Tables

**Table 1:** Pathologies induced by mitochondrial dysfunction.

Mitochondrial Pathologies	References
Aging, age-related diseases. Chronic inflammation	Douglas et al., 2011 [7]
Insulin resistance, type 2 diabetes, obesity, cardiovascular diseases, stroke	Bhatti et al., 2017 [8]
Neurodegenerative diseases, cancer	Thanan et al., 2015 [9] Verschoor et al., 2013 [10]
Respiratory diseases	Natarajan et al., 2014 [11]

**Table 2:** Pathologies with mitochondrial dysfunction as possible ozone indications.

Pathology and Type of Study	Procedure	Oxidative Stress/Repair Parameters Here Discussed	References
Chronic inflammatory processes			
Knee osteoarthritis			
Preclinical trial in rats. Effect of intra-articular ozone application on redox status in experimentally induced arthritis. Preclinical trial	6 groups, n = 48. 5–6 mL rectal ozone application; 0.5–0.7–1.0 mg/kg rat (week 1 to 3), 15 applications in prevention, 10 in the therapeutic group	Ox stress: MDA Repair: GSH	Mawsouf, N. et al., 2011 [12]
Ozone + Arthroscopy: Improved redox status, function and surgical outcome in knee osteoarthritis patients Clinical trial	40 patients. Group 1, n = 20, surgical procedure by arthroscopy. Group 2, n = 20, preventive ozone + arthroscopy. Rectal ozone insufflation. conc. 25–35 µg/mL, vol. 150–200 mL increasing in 4 weeks, 5 × /weekYes	Ox stress: MDA Repair: GSH GGT Beginning after ozone and 30 days after ozone + arthroscopy, or arthroscopy alone	León Fernández et al., 2020 [13]
Articular ozone modulates inflammation and has anabolic effect on knee osteoarthritis: IL-6 and IGF-1 as pro-inflammatory and anabolic biomarkers. Clinical trial	51 patients (non-diabetic, non-obese). 4 weeks, 1 × /week, intra-articular 20 mL, conc. 20 µg/mL	Pro-inflammatory cytokine IL-6	Fernández-Cuadros et al., 2022 [14]
Rheumatoid arthritis			
Medical ozone increases methotrexate clinical response and improves cellular redox balance in patients with rheumatoid arthritis Clinical study	60 patients, MTX group (n =30) basic therapy; MTX group with basic therapy (methotrexate + ibuprofen + folic acid). Ozone group (n = 30): treatment in the same way as the MTX group + ozone. Ozone treatment: 20 rectal insufflations at 25–40 µg/mL, 150–200 mL during 4 weeks	Ox stress: MDA Repair: GSH	León et al., 2016 [15]
Medical ozone effects and innate immune memory in patients with rheumatoid arthritis treated with methotrexate + ozone after a second cycle of ozone exposure. Clinical study	This study shows the same results as revealed in [15]; even better after a second cycle of ozone treatment due to the formation of memory cells during or after the first treatment cycle	Ox stress: MDA Repair: GSH	Takon-Oru et al., 2019 [16]
Medical Ozone: A redox regulator with selectivity for patients with rheumatoid arthritis. Clinical study	Although RA and osteoarthritis are of different origin, both are compared concerning the impact of redox regulation through ozone as usual; see above	Ox stress: MDA Repair: GSH	León Fernández et al., 2024 [17]
Diabetes			
Ozone treatment reduces markers of oxidative and endothelial damage in an experimental diabetes model in rats Preclinical study	Preclinical trial with STZ (Streptozotocin) induced diabetes in rats, n = 50. As type 2 diabetes is closely associated with oxidative stress, this animal model confirms the hypothesis that medical ozone is able to regulate and/or restore redox balance. Procedure: rectal insufflation with an ozone amount of 1.1 mg/kg rat, 10 treatments in 2 weeks	Ox stress: MDA Repair: GSH	Al Dalain et al., 2001 [18]
Therapeutic efficacy of ozone in patients with diabetic foot	Clinical study in patients with diabetes and diabetic gangrene. Antibiotic group, n = 49, received antibiotic infusions according to the specific germs. Ozone group, n = 51, 20 rectal ozone insufflations, 5 per week at 200 mL, conc. 50 µg/mL. Healing process improved; plasma glucose decreased. Oxidative stress decreased corresponding to an increase in SOD	GSH and SOD as repair parameters. TH as oxidative stress parameter	Martínez-Sánchez et al., 2005 [19]
Cancer			
Modulation of oxidative stress by ozone therapy in the prevention and treatment of chemotherapy-induced toxicity: review and prospects	Improvement in clinical results. Further trials necessary	Review: No redox data available	Clavo et al., 2019 [20]
Cancer. Complementary concept, head and neck tumors	Comparative study. N = 19	Clinical data; no redox data available	Clavo et al., 2019 [21]
Radiation-induced rectal bleeding in prostate cancer	12 patients successfully treated by rectal ozone insufflation	No redox data available	Clavo et al., 2015 [22]
Protection by ozone preconditioning in cisplatin-induced nephrotoxicity in rats Preclinical study	Rectal insufflation, conc. 20 + 30 µg/mL, 15 applications over 3 weeks prior to cisplatin. 10 groups with 7 animals each; here, cisplatin group as control and 2 ozone groups	Ox stress: MDA Repair: GSH	Borrego et al., 2004 [23]
Aging			
Ozone ameliorates age-related oxidative stress changes in rat liver and kidney: effects of pre- and post-aging administration. Animal trial	Ozone Group: rectal ozone application regularly from month 4 to month 14 (3 × per week; later, 2 × per week; ozone concentration: 20 mg/L; volume approx. 5 mL corresponding to 0.6 mg/kg body weight)	Ox stress: MDA Repair: GSH	Safwat et al., 2014 [24]
Medical ozone arrests oxidative damage progression and regulates vasoactive mediator levels in elderly patients (60–70 years) with oxidative etiology diseases Clinical study	20 rectal ozone insufflations, 5 per week at 200 mL, conc. 20 to 30 µg/mL	Ox stress: MDA Repair: GSH	León Fernández et al., 2022 [25]

**Table 3:** Redox markers, which are used to determine the redox status in the biological system. For the sake of understanding, we will essentially limit ourselves here to MDA and GSH; in one case, each TH as oxidative stress parameters, SOD as antioxidant and IL-6 as pro-inflammatory cytokine (bold). Preclinical or clinical trials where stress or repair markers are not available are not evaluated (↓:decrease; ↑: increase; ↑ ↑: remarkable increase).

Oxidative Stress Markers	Protective Stress Markers, Antioxidants	Cytokines
**TH** ↓ total hydroperoxides	Total **SOD** ↑ superoxide dismutase	Interleukins IL-1, **IL-6** ↓ TNF-α ↓ Tumor necrosis factor IFN-γ↑
**MDA** ↓ malondialdehyde	**GSH** ↑ ↑ reduced glutathione	
NO ↓ nitrogen oxide	γ-GT ↓ γ glutamyltransferase	

## Data Availability

Data are contained within the article.
